# Validation of the Ottawa knee rule in adults: A single centre study

**DOI:** 10.1002/jmrs.411

**Published:** 2020-06-21

**Authors:** Jordan I. Sims, Minh Chau, Josephine Davies

**Affiliations:** ^1^ UniSA Allied Health and Human Performance University of South Australia 108 North Terrace Adelaide South Australia 5001 Australia; ^2^ Medical Imaging Department Flinders Medical Centre Flinders Drive Bedford Park South Australia 5042 Australia

**Keywords:** Knee injuries, radiography, clinical decision rules

## Abstract

**Introduction:**

This clinical audit aimed to evaluate performance of the Ottawa Knee Rule (OKR) and degree of compliance by emergency referrers for acute knee injuries in adults.

**Methods:**

Knee radiography requests were analysed retrospectively for eligibility. Data were extracted from eligible requests under headings describing the OKR criteria, patient history, diagnosis and referrer profession. Sensitivity, specificity, negative likelihood ratio and positive likelihood ratio were calculated with 95% CI for the entire sample and each profession (consultant doctors, resident medical officers [RMO], physiotherapists and triage nurses) individually. The frequency of each OKR criterion and correlation with fracture, referrer compliance to the rule and the relative reduction in radiography were also calculated.

**Results:**

Of 713 patients identified, 149 were enrolled by the eligibility criteria. The overall sensitivity, specificity, negative likelihood ratio and positive likelihood ratio of the OKR for knee fracture were 71% (95%CI, 49‐87%), 46% (95%CI, 37‐55%), 0.64 (95%CI, 0.33‐1.22) and 1.3 (95%CI, 0.96‐1.76), respectively. Physiotherapists and triage nurses demonstrated better rule performance than consultant doctors and RMOs, with a sensitivity of 100% and negative likelihood ratio of 0.0. Physiotherapists were most compliant at 73% (19/26). Only 85 requests were OKR positive and, when abiding by the rule, this would have reduced radiography by 43% (64/149).

**Conclusions:**

In this first Australian study, moderate OKR performance and variable compliance by emergency referrers were observed. This led to unnecessary irradiation of patients without a fracture. The findings suggest emergency referrers could benefit from education on applying and documenting the OKR on radiography requests.

## Graphical abstract

Moderate accuracy and variable compliance with the Ottawa Knee Rule (OKR) was observed in this study. The overall sensitivity, specificity, negative likelihood ratio (LR‐) and positive likelihood ratio (LR+) of the OKR for knee fracture were 71% (95% CI, 49 to 87%), 46 (95% CI, 37 to 55%), 0.64 (95% CI, 0.33 to 1.22) and 1.3 (95% CI, 0.96 to 1.76), respectively. Physiotherapists were most compliant at 73%, and the relative reduction in radiography was 43% (64/149).

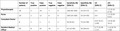

## Introduction

Acute knee injury is a common presentation in the emergency department and accounts for a significant use of plain radiography.^1^, ^2^ As per the American College of Radiology Appropriateness Criteria, plain radiography is the standard diagnostic tool for establishing bony injury.^3^ The Ottawa Knee Rule (OKR) was derived in 1995 to provide a clinical decision aid for emergency referrers in ruling out knee fractures.^2^ The rule assesses five criteria: age 55 years or older, tenderness of fibular head, isolated tenderness of patella, inability to flex to 90 degrees and inability to weight bear for 4 steps immediately after injury and in the emergency department (ED).^2^ Patients with positive OKR findings are highly likely to have sustained a fracture and subsequently require radiographic investigation.^2^

Several studies have proven the value of OKR in knee fracture assessment, with a sensitivity approaching 100%.^4^, ^5^, ^6^, ^7^, ^8^ A recent systematic review and meta‐analysis by Sims, Chau and Davies demonstrated the rule to have a sensitivity of 99% and negative likelihood ratio of 0.07.^9^ Results of the review by Bachmann and colleagues were similar, with a sensitivity of 98.5% and negative likelihood ratio of 0.05.^10^ This alone boosts the efficiency of patient care in ED and limits the costs of unnecessary radiography and radiation exposure.^1^, ^2^, ^4^, ^11^ Other decision guidelines exist, such as the Pittsburgh Rule^12^ and those by Weber^13^ and Bauer and colleagues^14^, but do not possess this degree of validation in the literature. However, referrer compliance to the OKR has been described as poor due to several patient, medico‐legal and confidence barriers.^1^, ^4^, ^11^, ^15^, ^16^ The generalisability of results is also limited, particularly in Australia. To our knowledge, no audit has been conducted in Australia which evaluates performance of the OKR and referrer compliance to the rule without prior formal training.

The primary objectives of our audit were to assess consultant doctor, resident medical officer (RMO), physiotherapist and triage nurse compliance with OKR and calculate rule performance for each profession audited. The secondary objective of this study was to determine whether use of the rule would reduce the number of knee radiographs ordered.

## Methods

### Ethics

The research study was reviewed and given exemption by both the Southern Adelaide Local Health Network and University of South Australia Human Research Ethics Committees.

### Study design and setting

A retrospective audit was conducted at a tertiary hospital in South Australia, where patients are referred for radiography typically by consultant doctors, RMOs, physiotherapists and triage nurses. At our centre, triage nurses trained in NIXR (nurse‐initiated X‐ray) and physiotherapists with appropriate training and qualifications may request radiography. For the purposes of this study, RMOs included emergency registrars and excluded medical interns and students. Eligible patients and reports were identified using the Picture Archiving and Communication System (PACS), and the Radiology Information System (RIS) was used to document the profession of the referrer.

### Participants

Our study population consisted of patients 18 years of age and older who presented with acute knee trauma to the institution’s emergency department in an 8‐month period between May 2019 and December 2019.

### Procedure

All emergency requests for knee radiography were screened for eligibility, and all ineligible requests were categorised based on the exclusion criteria; paediatric patient, multi‐trauma or multiple areas requested, injury occurred over 7 days prior to presentation, relevant pre‐existing disease or previous injury, and no history of trauma. As it was an acute setting, the study did not identify or include any requests for follow‐up radiography of confirmed fractures. Data were extracted from the requests into a Microsoft Excel spreadsheet (Microsoft Office 16). Headings included the specific OKR (including five criteria), patient history, patient ID, date of birth, gender, date, examination, referrer profession (consultant doctors, RMOs, physiotherapists or triage nurses) and diagnostic outcome.

### Data analysis

For our primary objective, compliance of the referrer with the rule was assumed if at least one of the criteria was met or, if none were met, the referrer specifically indicated a negative OKR result. This was expressed as a percentage of the total requests completed by the referrer. Evidence of other decision rules was noted separately from the OKR. The performance of the rule for identifying patients with a fracture was examined in the study cohort by calculating sensitivity, specificity, positive likelihood ratio (LR+) and negative likelihood ratio (LR‐) with 95%CI. This was performed by constructing 2x2 contingency tables for the entire sample and for each profession individually. As the OKR is solely used to rule out fracture and is therefore not a definitive diagnostic tool, we did not calculate diagnostic accuracy. For our secondary objective, relative reduction in radiography was calculated as the difference in the total sample size and the number of OKR positive cases. The documented frequency of each OKR criterion and its associated fracture correlation was also calculated (%).

## Results

**Figure 1:**
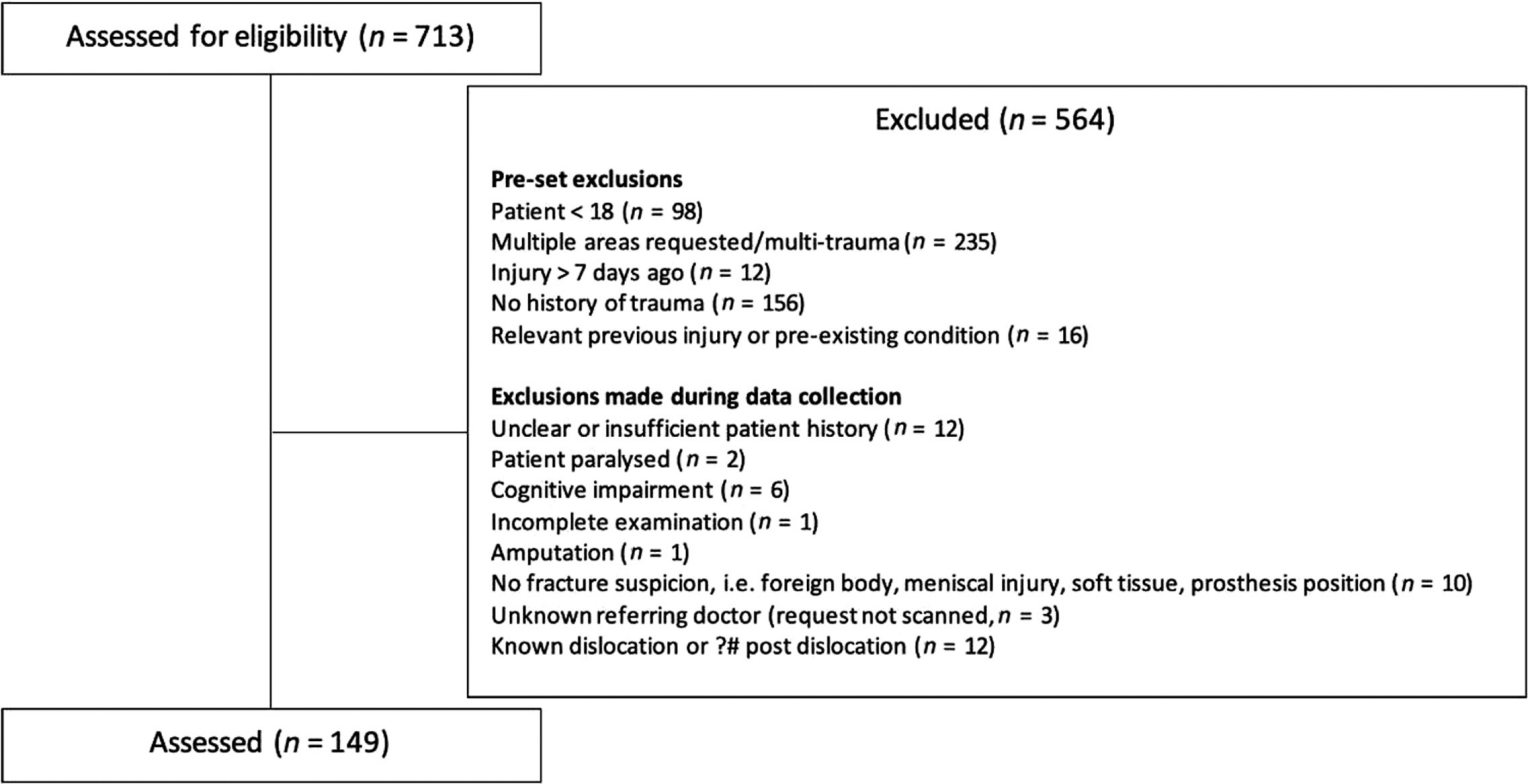
Patient selection criteria.

A total of 713 knee radiography requests were gathered from May to December 2019. Of these, 149 met the inclusion criteria (20.9%). Our pre‐set criteria (adult, localised injury to knee, injury within 7 days, no relevant pre‐existing disease or injury, and no history of trauma), and additional exclusions determined during data collection, are outlined in Figure 1. The included age range was 18‐93Y, with a mean age of 44.29. Approximately half (48.9%) of the patients were female.

### Overall performance of the OKR

Of the 149 patients, 24 had a confirmed knee fracture, and thus, the prevalence of fracture was 16.1% (24/149). The 2x2 contingency table for all patients in our study is outlined in Table 1. Overall, the sensitivity, specificity, LR‐ and LR + of the OKR for knee fracture were 71% (95%CI, 49‐87%), 46% (95%CI, 37‐55%), 0.64 (95%CI, 0.33‐1.22) and 1.3 (95%CI, 0.96‐1.76), respectively.

**Table 1:** Diagnostic outcome of all patients included in analysis.

	Fracture	No fracture	Total
Rule positive	17 True positive	68 False positive	85
Rule negative	7 False negative	57 True negative	64

Of the five OKR criteria, age over 55 was the most commonly documented at 36.9% (55/149). Patella tenderness was reported for 18.8% (28/149), inability to weight bear for 18.1% (27/149), inability to flex to 90 degrees for 4.7% (7/149) and fibular head tenderness for 3.4% (5/149) of patients. 18.5% (5/27) of patients unable to weight bear, 28.6% (2/7) of patients unable to flex to 90 degrees, 14.3% (4/28) of patients with patella tenderness, 40% (2/5) of patients with fibular head tenderness and 25% (14/55) of patients over 55 had fractures.

### Profession‐specific performance of the OKR

A total of 26 referrals were completed by physiotherapists, 20 by triage nurses, eight by consultant doctors and 95 by RMOs. Physiotherapists and nurses performed best, with a sensitivity and LR‐ of 100% and 0.0, respectively. No false‐negative results were noted for these professions, while five were seen for RMOs and two for consultant doctors. Table 2 summarises the specific results for each profession.

**Table 2:** Performance results of Ottawa Knee Rule for each emergency referrer group.

	Number of patients	True positive	False positive	True negative	False negative	Sensitivity (%) [95% CI]	Specificity (%) [95% CI]	LR‐[95% CI]	LR+[95% CI]
Physiotherapist	26	5	14	7	0	100 [46‐100]	33 [15‐57]	0.0	1.5 [0.16‐0.79]
Nurse	20	2	12	6	0	100 [20‐100]	33 [14‐59]	0.0	1.5 [0.05‐0.61]
Consultant Doctor	8	2	1	3	2	50 [9‐91]	75 [22‐99]	0.67 [0.21‐2.09]	2.0 [0.28‐14.2]
Resident Medical Officer	95	8	41	41	5	62 [32‐85]	50 [39‐61]	0.77 [0.38‐1.57]	1.23 [0.76‐1.99]

### Compliance with the OKR

Compliance between the professions with the OKR was variable. 73% (19/26) of the requests completed by physiotherapists and 65% (13/20) by nurses demonstrated compliance. The compliance for consultant doctors and RMO requests was 37.5% (3/8) and 48.4% (46/95), respectively. Moreover, 35% (9/26) of physiotherapist requests and one nurse request utilised the rules by Bauer and colleagues,^14^ namely medial or lateral ‘joint line tenderness’. In five cases, RMOs used these rules and four cases demonstrated evidence of both Bauer and colleagues and OKR.

### Relative reduction in radiography

The secondary objective of this study was to determine whether use of the OKR would reduce the number of knee radiographs ordered for adults presenting with knee injuries to the ED. If abiding by the OKR, only requests positive for the rule require radiography. Of the 149 eligible requests, 85 were OKR positive and this would have resulted in a 43% (64/149) reduction in radiography.

## Discussion

Since the development of the OKR in 1995,^2^ its excellent sensitivity (near 100%) has been argued and found indisputable across many countries.^1^, ^4^, ^5^, ^6^, ^7^, ^8^, ^17^ However, few published studies have investigated the local generalisability of the rule, particularly in Australia. Our retrospective clinical audit evaluated OKR performance and referrer compliance in a tertiary hospital in South Australia. Our results demonstrate the OKR to have an overall sensitivity, specificity, LR‐ and LR + of 71% (95%CI, 49‐87%), 46% (95%CI, 37‐55%), 0.64 (95%CI, 0.33‐1.22) and 1.3 (95%CI, 0.96‐1.76), respectively. In general, physiotherapy and nursing professions performed best, both demonstrating 100% sensitivity and 0.0 LR‐. This indicates a positive OKR has a high probability of identifying a fracture and a negative OKR has good odds for the absence of fracture. Requests completed by physiotherapists and nurses also demonstrated 73% and 65% compliance with the rule, respectively. Consultant doctors and RMOs showed poorer performance of the rule, with sensitivities and LR‐ less than 65% and 0.8, respectively. Particularly, the false negatives (n = 7) and lower compliance of 37.5% for consultant doctors and 48.4% for RMOs were unpromising. Had they utilised the OKR to justify radiography, such false‐negative results would misdiagnose patients and risk potential for further injury. According to the present study where 85 requests were OKR positive, implementing the OKR would have resulted in an overall 43% reduction in radiograph use (64/149). Nevertheless, the true reduction in radiographs cannot be established unless a prospective study or an implementation trial is performed with follow‐up of patients without radiography. Follow‐up is essential because in spite of the fact that the radiologist was a board‐certified and experienced practitioner, it is still possible that some fractures were missed. The implementation trial could also involve the interpretation of two independent musculoskeletal radiologists to ensure a more robust methodology.

In general, our findings propose moderate performance of the OKR for identifying a knee fracture. Unsurprisingly, this is much lower than previous studies, as the majority of methodologies included formal training in the OKR and solely evaluated medical officers. In their systematic review, Bachmann and colleagues (2004) demonstrated the rule to be 98% sensitive and 49% specific for knee fracture.^10^ However, Atkinson and colleagues, who investigated radiography requesting patterns prior to teaching the OKR, observed only 80% sensitivity.^18^ This study confirms our results and, together, suggests poor referrer awareness of the rule. In our study, the 43% potential reduction in radiography if the OKR was correctly applied is consistent with studies performed in Canada, Iran and Spain, which calculated reductions of 31.2%, 41% and 49%, respectively.^6^, ^17^, ^19^ Our value is, however, higher than the rate in the original report by Stiell and colleagues (26.4%).^1^ Finally, although the patient’s time spent in ED was not specifically examined in our study, using the OKR could shorten the waiting time for our patients in ED. The original publications of the rule^1^, ^2^ found that adults who underwent knee radiography spent an average of 127 minutes in ED compared to 83 minutes for those who did not need radiography, and similar results were calculated in the implementation trial.^1^, ^2^

As with any study, there are several opportunities for bias to impact the results. In our measures of compliance, no written evidence of rule application was assumed ‘non‐compliant’. It is possible, however, referrers unknowingly acknowledged the OKR and still requested radiography. If so, the indicator for requesting radiography remains unknown. Furthermore, as each referrer requested radiography independently, we could not assess interobserver agreement for rule application. Given our retrospective methodology, we do not predict this to have introduced poor reliability. Unlike previously established methodologies, we did not limit our study to clinically important fractures (>5mm fracture length) and included all bony injuries, which may account for the seven false‐negative results. Furthermore, with a restricted timeframe (8 months) and extensive exclusions list, we did not include all patients presenting with acute knee injury and achieved only a small sample size (n = 149). This led to uneven distribution of patients between referrers and may be the cause of the high fracture prevalence in our sample (16.1%) when compared to other studies.^1^, ^4^, ^5^, ^6^, ^7^, ^8^ The high prevalence may also be attributed to the nature of the hospital as a tertiary centre. Although some referrers utilised the decision rules by Bauer and colleagues,^14^ this was less commonly observed in our study than the OKR. Another decision guideline, the Pittsburgh rule, consists of two criteria identical to the OKR (over 55 years of age, inability to weight bear),^5^ and it was therefore impossible to ascertain whether this was applied instead of or in conjunction with the OKR. Hence, our estimates of compliance may be positively skewed ‘pro’ OKR. Finally, as this was only a clinical audit, we could not definitively comment on the causes of performance discrepancy between the different professions. All referrers were not made aware of this quality improvement study, and documentation of the use of OKR on the request was not required. We suspect that consultant doctors and RMOs subconsciously used the OKR but did not document on the request form due to time constraints, workload and ambivalence about whether the radiologist/radiographer would require a full documentation of OKR. Additionally, the sample sizes for consultant doctors (8) and RMOs (95) are not consistent with the sample sizes for physiotherapists (26) and nurses (20). This might reflect an unbalanced result. We also predict the specialist training available to nurses and physiotherapists for requesting radiography would include clinical decision rules. To fully understand the principles behind these discrepancies, further qualitative research studies are required.

Considering the low rates of compliance, we recommend a local survey of all emergency referrers to gauge the level of OKR awareness and the referrer‐perceived barriers to application. Analysis of the compliance rates within a single profession could also be performed to correlate level and/or recency of training with rule awareness. Using this information, a formal training model should be developed to educate referrers, improve compliance and reduce radiography requests. Hospitals should also investigate installing decision support tools and prompts on computer systems, as suggested by Beutel and colleagues.^15^ Re‐auditing post‐intervention is advised. Future implementation techniques may also involve educating radiographers on the OKR to assist their assessment of unjustified requests.

## Conclusion

Although the OKR has been validated internationally, this is the first study to investigate its performance and referrer compliance in Australia. Our audit demonstrated moderate rule performance and variable compliance between the emergency referrers and the OKR. As a result, patients presenting to this centre received unnecessary radiation exposure. When implemented appropriately, the OKR can effectively rule out knee fractures and reduce patient waiting time in the emergency department. Hence, the findings of this study indicate that all emergency referrers could benefit from local education on how to apply and document OKR in radiography requests. Periodic audits to monitor compliance are also recommended.

## Conflict of interests

The authors declare no conflicts of interest.
